# TNBC Challenge: Oligonucleotide Aptamers for New Imaging and Therapy Modalities

**DOI:** 10.3390/ph11040123

**Published:** 2018-11-13

**Authors:** Simona Camorani, Monica Fedele, Antonella Zannetti, Laura Cerchia

**Affiliations:** 1 Istituto per l’Endocrinologia e l’Oncologia Sperimentale G. Salvatore (IEOS), CNR, 80145 Naples, Italy; s.camorani@ieos.cnr.it (S.C.); mfedele@unina.it (M.F.); 2 Istituto di Biostrutture e Bioimmagini, CNR, 80145 Naples, Italy; antonella.zannetti@cnr.it

**Keywords:** aptamer, chemoresistance, targeted imaging, targeted therapy, TNBC, tumor microenvironment, SELEX

## Abstract

Compared to other breast cancers, triple-negative breast cancer (TNBC) usually affects younger patients, is larger in size, of higher grade and is biologically more aggressive. To date, conventional cytotoxic chemotherapy remains the only available treatment for TNBC because it lacks expression of the estrogen receptor (ER), progesterone receptor (PR) and epidermal growth factor receptor 2 (HER2), and no alternative targetable molecules have been identified so far. The high biological and clinical heterogeneity adds a further challenge to TNBC management and requires the identification of new biomarkers to improve detection by imaging, thus allowing the specific treatment of each individual TNBC subtype. The Systematic Evolution of Ligands by EXponential enrichment (SELEX) technique holds great promise to the search for novel targetable biomarkers, and aptamer-based molecular approaches have the potential to overcome obstacles of current imaging and therapy modalities. In this review, we highlight recent advances in oligonucleotide aptamers used as imaging and/or therapeutic agents in TNBC, discussing the potential options to discover, image and hit new actionable targets in TNBC.

## 1. Introduction

**Figure 1:**
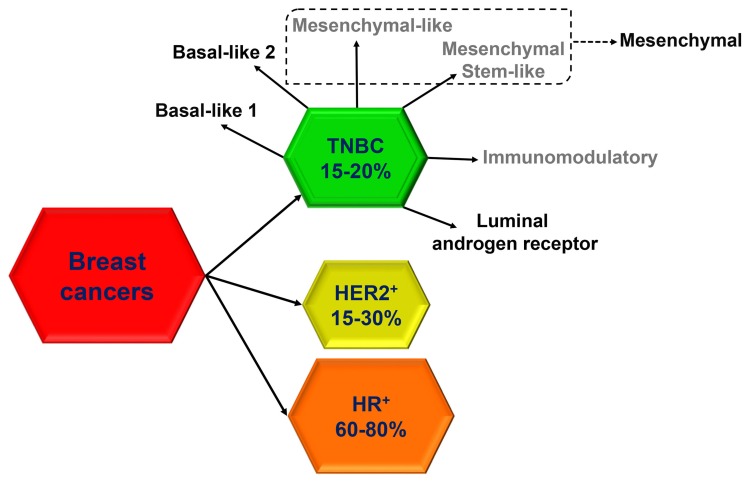
Heterogeneity of TNBC. Six distinct TNBC subtypes were identified by Lehmann et al. [3] including Basal-like 1, Basal-like 2, Mesenchymal-like, Mesenchymal Stem-like, Immunomodulatory and Luminal androgen receptor. This classification was later refined, by the same authors [4], into four subtypes (Basal-like 1, Basal-like 2, Mesenchymal and Luminal androgen receptor). See text for details. The percentage of TNBC [1], HER2-positive [5] and hormone receptor (HR)-positive [6] breast cancers are reported.

Triple-negative breast cancer (TNBC), which accounts for 15% to 20% of breast cancers, more frequently affects younger patients, is more prevalent in African American women and has frequently a poor prognosis [1]. Molecularly, TNBC is defined by the lack of receptors for estrogen (ER), progesterone (PR) and epidermal growth factor receptor 2 (HER2). However, defining TNBC through the absence of biological markers is limiting because of the high heterogeneity of this group of cancers, which include different subtypes according to unique histological and molecular characteristics, as well as to a distinct natural history and responsiveness to treatment. In the last decade, with the advent of ‘omics’ studies, many subtype classifications for TNBC have been proposed based on molecular profiles and clinical outcome, but, to date, none of the newly developed molecular classifications has demonstrated any clinical utility. From a histological point of view, most of TNBC (95%) are classified as invasive ductal carcinomas, while more rarely they include invasive lobular carcinomas (1–2%) or, in less than 1%, metaplastic carcinomas with squamous differentiation, spindle-cell metaplastic carcinomas, adenoid cystic carcinomas, secretory carcinomas, typical medullary carcinomas, atypical medullary carcinomas and apocrine carcinomas [2]. According to the 2011 molecular classification by Lehmann et al. [3], based on gene expression patterns, six distinct biological TNBC subtypes, including two basal-like (BL1, BL2), two mesenchymal-like (ML, MSL), one immunomodulatory (IM) and one luminal androgen receptor (LAR), were identified (Figure 1).

Different clusters of genes can be distinguished inside the TNBC subtypes. The first cluster, highly sensitive to anthracycline, is characterized by a signature of DNA repair genes, including loss of tumor protein 53 (TP53), retinoblastoma 1 (RB1), and BRCA1 function and enrichment in DNA damage response, cell proliferation, TP63, epidermal growth factor receptor (EGFR) and MET signaling [7,8]. The second cluster, the claudin-low breast cancer subtype, is characterized by mesenchymal features, low expression of cell-cell junction proteins (i.e., claudin, E-cadherin), intense immune infiltrate and bad prognosis [9,10]. The gene signature for this cluster also include enrichment in Wnt, transforming growth factor β (TGFβ), IGF1R, Notch, mitogen-activated protein kinase (MAPK), Rac, phosphatidylinositol 3-kinase (PI3K), platelet-derived growth factor (PDGF) and cell proliferation signaling pathways [7]. The third cluster, characterized by high expression of an immune response gene module and lymphocyte infiltration, is associated with better outcome among patients with TNBC [11,12], while the fourth and last cluster, characterized by androgen receptor (AR), forkhead box protein A1 (FOXA1) and ERBB4 expression and signaling, share common gene profile with ER-positive breast cancer [13]. More recently, Lehmann et al. refined their classification (Figure 1) and reduced the original six subtypes to four (BL1, BL2, mesenchymal and LAR) because they found that the IM and MSL subtypes are, indeed, contributed from infiltrating lymphocytes and tumor-associated stromal cells, respectively [4].

In this review, in the light of the current unsatisfactory scenario in the therapy and imaging of TNBC, we discuss recent experimental approaches to generate aptamers against TNBC cells with effective cell-type targeting and discriminating properties, which have great potential for discovery of new actionable biomarkers. Additionally, we highlight aptamer-based strategies to validate, as possible targets for therapeutic and/or imaging modalities, proteins with a recently proposed role in TNBC, including markers overexpressed on cancer cells, cancer stem cells (CSCs), stromal cell types within the TNBC microenvironment, as well as components of extracellular matrix (ECM).

## 2. Current Diagnostic Imaging and Therapy of TNBC

Despite the great progress in the identification and molecular characterization of different TNBC subtypes, the imaging role in TNBC management is not yet well defined. The first-line diagnostic modalities such as ultrasound and mammography are not the best tools for initial evaluation of TNBC because of the possibility of false negative results due to imaging features similar to benign breast tumors, which are characterized by the absence of calcifications and well-defined margins [14]. However, Moon et al. [15] reported that a computer-aided diagnosis system, based on texture features extracted via the Ranklet transformation, may be advantageous to differentiate TNBC from benign fibroadenomas. Furthermore, a recent retrospective study on a large patient cohort with fibroadenomas or TNBC, using a radiomics score based on ultrasound texture analysis demonstrated a high differential diagnostic value [16]. Nowadays, the contrast-enhanced breast magnetic resonance imaging (MRI) represents the gold standard in imaging modality to predict histological diagnosis and prognosis of TNBC. Indeed, Angelini et al. confirmed that TNBC is commonly associated with higher T2 signal intensity, rim enhancement and unifocality in MR images than receptor positive breast carcinomas [17,18]. Dynamic contrast-enhanced MRI features, showing the presence of a concentric shrinkage pattern in TNBC patients after neoadjuvant chemotherapy (NAC), are associated with a pathologic complete response (pCR) [19]. Furthermore, Groheux et al. [20] showed that the early changes in 18F-fluorodeoxyglucose (18F-FDG) uptake assessed by positron emission tomography-computed tomography (PET/CT) could be useful to predict pCR to NAC and patient outcome. However, all these imaging techniques need to be validated in larger trials. The progress in multi-modality imaging as well as in radiomic analysis will surely improve the diagnosis, staging and management of the TNBC subtypes. Recently, it has been reported that simultaneous use of diffusion-weighted MRI combined with 18F-FDG-PET/CT, in cisplatin-treated TNBC tumor-bearing mice, could differentiate between responders and non-responders predicting therapeutic response [21]. Even if it is well known that each TNBC subtype shows different clinical outcome and response to treatment, to date there are no imaging modalities able to discriminate between them. Therefore, it is of high-priority to identify clinically applicable biomarkers that can help in validating a TNBC diagnosis and guiding the personalized treatment.

Currently, no targeted therapies have been approved for TNBC yet, but cytotoxic chemotherapy remains the standard treatment for patients in both the early and advanced stages. A variety of nonspecifically designed agents is used for therapy, including taxanes (paclitaxel, docetaxel), anthracyclines (e.g., doxorubicin, epiribucin), alkylating agents (cyclophosphamide), antimetabolites (methotrexate) and platinums (cisplatin) [1]. However, although some patients respond, the treatment is toxic, and a large percentage of tumors treated in the early stage eventually relapse causing a death rate in the metastatic disease disproportionately higher than any other subtypes of breast cancer [7].

A few biomarker-driven therapies have been so far proposed for TNBC beyond conventional chemotherapy, including inhibitors of immune-checkpoints, PI3K-Akt-mTOR and MAPK pathways, EGFR, as well as either poly-ADP ribose polymerase inhibitors or AR antagonist for TNBC harboring BRCAness and LAR subtypes, respectively. Novel therapeutic strategies for TNBC have been recently reviewed in detail [22].

## 3. SELEX Technology

SELEX technology, described for the first time in 1990 [23,24,25], has been increasingly applied to different areas of cancer medicine, including biomarker discovery, biosensing, bioimaging and therapy. Through SELEX, highly structured, short, single-stranded (ss) DNA or RNA oligonucleotides, called aptamers, are selected to bind to a chosen target with high affinity and specificity (Kd values in the low nanomolar to picomolar range), in a manner similar to antibody-antigen interactions. Aptamers have been proved as a valid alternative to antibodies as activating ligands, inhibitors or carriers of either therapeutic or imaging agents [26,27]. 

SELEX is a multistep process in which random libraries of 10^13^–10^15^ ssDNA or RNA oligonucleotides, undergo repetitive cycles of: (i) incubation with the target; (ii) partitioning of the target-bound from unbound sequences; (iii) recovery and amplification of the bound sequences, to obtain a progressive enrichment for high affinity binding oligonucleotides. After a sufficient number of cycles, individual sequences are identified by cloning of the final selected pool or high throughput Next-Generation Sequencing (NGS) technology and subsequent bioinformatic analysis [28,29], and tested for affinity and selectivity. Over the years, numerous protocols of the SELEX process have been developed focused to accelerate the selection process and enhance its success rate [28]. Despite their differences, most selection methods include counter-selection steps against sham targets to avoid enrichment for aptamers against unwanted targets and improve aptamers selectivity for the chosen target. To date, SELEX allowed to select thousands of aptamers against a wide range of targets, including small molecules [30], proteins [31], whole live cells [32], tissues [33], and even targets within live animals [34,35,36].

**Figure 2:**
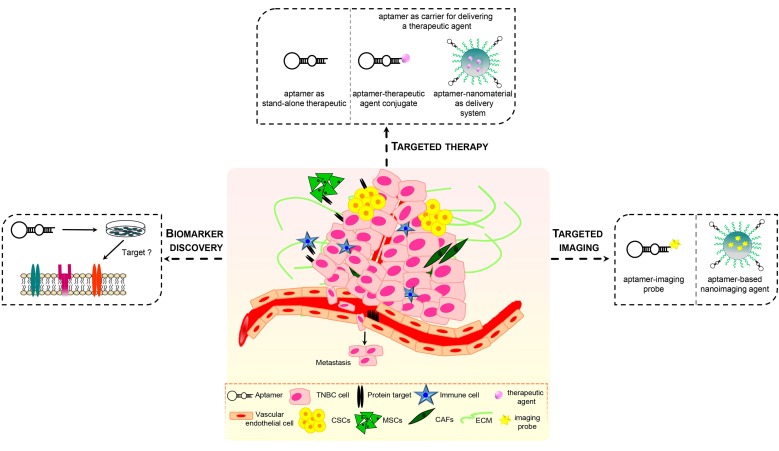
Applications of aptamers in TNBC. Aptamers against targets, which have a crucial role in TNBC and surrounding microenvironment, may serve as therapeutics, by modulating target’s function or delivering other therapeutic agents (chemotherapeutics, small therapeutic oligonucleotides), imaging agents and targeting agents for biomarkers discovery.

Recently, aptamers have been used as efficacious recognition elements for TNBC cells. As discussed below, because of their nucleic acid nature, aptamers may be manipulated, easier than antibodies, for both diagnostic and therapeutic applications thus showing a strong potential for: (1) identifying or validating novel actionable targets; (2) interfering with the activity of the target; (3) delivering imaging agents, chemotherapeutics and nanoparticles specifically into affected cells (Figure 2). Notably, aptamer-based targeted molecular strategies have the potential to overcome current obstacles for TNBC management mostly due to the high heterogeneity of these tumors, drug resistance and abnormal tumor microenvironment (TME).

## 4. Cell-SELEX against TNBC Cells to Generate Cell-Type Targeting Aptamers

The biological/clinical heterogeneity of TNBC and the lack of targeted therapies indicate the need to identify novel druggable targets for patient stratification and to develop targeted treatment approaches able to counteract each individual TNBC subtype. The pCR rate differs according to the high genetic diversity within TNBC subtypes indicating that a distinct molecular phenotype may dictate response to treatment. Accordingly, BL subtypes, showing enrichment of proliferation genes, have a better response to taxane-based therapies as compared to ML or LAR subtypes [37].

Because of their high specificity, aptamers able to distinguish between small differences in the cell surface protein signature of heterogeneous cancers may represent useful tools for molecular recognition and characterization of TNBC cells, paving the way to development of personalized treatments for these tumors. Although protein-SELEX, which uses a recombinant protein as target for the selection, has successfully generated many highly-specific cell-targeting aptamers, allowing for low non-specific binding and easy control of the selection conditions, cell-SELEX represents the most accurate choice to generate aptamers specific for cell-surface biomarkers. Indeed, using for the selection entire cells in culture, expressing a pre-identified target protein instead of the protein in a recombinant soluble form, cell-SELEX eliminates the risk of selecting aptamers failing to recognize the target in its natural conformation and distribution on the cell surface. This is crucial for transmembrane proteins and receptors that are usually glycosylated, may exist in different conformations and interact with neighboring proteins or the TME, to exert their effects on specific cellular processes. Obviously, cell growth and maintenance conditions are critical for successful cell-SELEX. Recently, SELEX protocols have been settled to eliminate dead cells during the process [38] and to select aptamers on spheroid cells in three-dimensional (3D) cell cultures resembling in vivo environments [39].

Importantly, cell-SELEX can be carried out against a specific cell type, without the prior knowledge of molecules present at the cell surface. Altering target cell-selection and off-target cell-elimination steps is fundamental to improve the selectivity of aptamers for the cell type used as target. In such a way, aptamers may identify subtle differences existing among different cells, even belonging to the same tumor type, which drive important tumor cell behaviors, including resistance to therapy, tumorigenicity, stemness and capacity to metastasize. Also, post-SELEX approaches are used for identification of the targets recognized by the best binding sequences, thus allowing for discovery of new critical biomarkers (Figure 2). Numerous cell-SELEX protocols have been to date developed for isolating aptamers with effective cell-type targeting and discriminating properties [40,41,42,43,44,45]. Recently, some approaches used human MDA-MB-231 cells, belonging to the ML TNBC subtype, as target of the positive selection because their highly malignant and invasive phenotype. Li et al. screened a DNA library on MDA-MB-231 cells to generate aptamers specifically discriminating target cells from the non-tumorigenic breast epithelial MCF-10A cells used in the counter-selection steps [46]. Almost all the selected aptamers were specific to metastatic MDA-MB-231 and T47D breast cancer cells, with no obvious binding to other types of cancer cells, such as cervical cancer cells (HeLa) and liver cancer cell lines (QGY-7703 and HepG2). Importantly, one aptamer, whose target has not yet been identified, once fluorescently labeled with a FAM dye and used for tissue imaging, was able to stain breast cancer tissue with metastasis in one or more regional lymph nodes at a higher rate (76% positive rate, among 34 tissue samples) with respect to breast cancer tissue with no regional lymph node (39% positive rate, among 38 tissue samples).

More recently, a SELEX approach, using MDA-MB-231 as the target cells and low-metastatic MCF-7 breast cancer cells for the negative selection, was developed for the capture of circulating tumor cells (CTCs) with a metastatic phenotype [47]. Among a panel of five DNA aptamers binding specifically to target cells, M3, which displayed the highest affinity (Kd = 45.6 ± 1.2 nM), was immobilized in a biotin-labeled form on streptavidin plates and used to capture the target cells spiked into non-target cells or CTCs in whole blood from metastatic breast cancer patients. Interestingly, it proved able to detect especially cells do not expressing the epithelial cell adhesion molecule (EpCAM), which is widely used as a marker for CTC enrichment.

In a proof of concept study [48], MDA-MB-231 cells were also used as target for a SELEX approach aimed to exploit artificial expanded genetic information systems (AEGIS-SELEX). The purpose of this approach was to screen a six-letter ssDNA library (containing the standard four nucleotides and two nonstandard nucleotides with a nitro functionality not found in standard DNA) on a cancer cell line in order to increase the power of SELEX by increasing sequence diversity. Even though a counter-selection was not added to the SELEX, and thus the resulting aptamers may be non-specific, this selection gave rise to aptamers with nanomolar dissociation constant against target cells.

Another important advantage of cell-SELEX is that it allows to select for cell-internalizing aptamers that may serve as selective intracellular delivery vehicles for diagnostic/therapeutic cargos upon binding to targets exclusively expressed and/or overexpressed on cancer cells and consequentially cell-internalization via receptor-mediated endocytosis [49,50].

To date, aptamers against cell surface proteins enabled targeted delivery in cancer cell lines of therapeutic small interfering RNAs (siRNAs), microRNAs (miRNAs) and anti-miRNAs [26], large functional RNAs [51], chemotherapeutics [52] and toxins [53], through either sophisticated direct conjugation to the cargo or diverse formulations of nanocarriers.

## 5. Aptamers Targeting Proteins Overexpressed on TNBC Cells

The diverse molecular subtypes of TNBC are characterized by the marked expression of certain biomarkers [3]. Although their presence is not restricted to TNBC, these molecules show increased prevalence in these tumors, also discriminating individual subtypes. As shown below, EGFR and PDGF receptor β (PDGFRβ) receptor tyrosine kinases (RTKs), mucin (MUC) and nucleolin (NCL) are among the targets which have been suggested to have a role in different aspects of TNBC behaviors, including vasculogenic mimicry (VM), metastasis and resistance to therapy. All these biomarkers have been targeted by aptamers in preclinical imaging and therapy of TNBC.

### 5.1. Aptamer Targeting EGFR and PDGFRβ Receptor Tyrosine Kinases

Transmembrane RTKs are key regulators of critical cellular processes, such as proliferation, differentiation, survival and migration. Upon binding to growth factors, neurotrophic factors, and other extracellular signaling molecules, RTKs undergo homo- or hetero-dimerization and tyrosine autophosphorylation resulting in the activation of downstream signaling pathways, including the PI3K/Akt and MAPKs. While their activity is tightly regulated under physiological conditions, aberrant activation of RTKs, by gene mutations and/or gene amplification and overexpression, occurs in most human cancers [54]. Unfortunately, clinical trials with RTK inhibitors, including monoclonal antibodies (mAbs) and small-molecule tyrosine kinase inhibitors (TKIs), showed disappointing outcome in patients with TNBC, which harbors multiple hyperactive RTKs but lacks receptor-activating mutations [3]. Recently, it has been reported that the inactivation of the PTPN12 tyrosine phosphatase, frequently observed in TNBC, leads to aberrant activation of MET, PDGFRβ, EGFR, and others RTKs thus suggesting a rational for combining RTK inhibitors in TNBC [55].

To date, several aptamers binding to human RTKs have been developed and explored to overcome some of the limitations of TKIs and mAbs, including limited efficacy and acquired resistance [56]. Among them, two aptamers developed by our laboratory, named CL4 and Gint4.T, which target EGFR and PDGFRβ, respectively, have been reported as promising candidates for therapy and imaging of specific TNBC subtypes [57,58,59]. Overexpression of EGFR has been reported in ~60% of TNBC and correlates with poor outcome [60]. By using the anti-EGFR 2′-Fluoro-pyrimidine (2′F-Py)-RNA CL4 aptamer, previously validated as an efficacious targeting agent in GBM [61], Her2-positive breast cancer and NSCLC [41], we demonstrated a novel mechanism of action for this aptamer related with integrin αvβ3-EGFR interaction in TNBC [57]. Indeed, it has been shown that cells of aggressive and poorly differentiated TNBC are able to trans-differentiate into endothelial-like cells thus forming vessel networks that increase tumor growth and metastasis with a mechanism distinct from classical angiogenesis and known as VM. We showed that, when ML TNBC MDA-MB-231 and BT-549 cells, expressing EGFR on their surface, are grown in 3D cultures or are injected in nude mice to form tumors, EGFR is associated with integrin αvβ3, one of the principal adhesion molecules expressed on cancer cells. This interaction allows integrin to adopt a conformation competent for binding to ECM and required for VM. The CL4 aptamer, when intravenously injected into mice bearing TNBC xenografts, prevented the formation of integrin αvβ3-EGFR complex, thus causing the inhibition of integrin attachment to matrix and, in turn, inhibition of VM and tumor growth [57]. Further, Shu et al. used the CL4 aptamer, which actively internalizes into target cells via receptor mediated endocytosis [61], to assemble RNA nanoparticles (NPs) consisting of the phi29 pRNA-three-way junction as a core, the CL4 as the targeting agent and the anti-miR-21 as therapeutic. These multifunctional vectors, intravenously injected into mice bearing orthotopic tumors derived from MDA-MB-231 cells, efficiently inhibited tumor growth [58].

It is well demonstrated that overexpression of PDGFRβ on endothelial cells and tumor-associated stromal cells occurs in different human cancers, where complex PDGFRβ-dependent signaling contributes to angiogenesis and tumor progression. More recently, this receptor has been found expressed on the surface of cancer cells belonging to most undifferentiated and aggressive human tumors, such as GBM, where it acts as a critical mediator of the stem-like phenotype [62]. Accordingly, the anti-PDGFRβ 2′F-Py-RNA Gint4.T aptamer was able to target PDGFRβ-positive GBM either subcutaneously or intracranially implanted in mice, acting as inhibitor of tumor growth [42] and delivery agent for drug-loaded nanoparticles [63]. In agreement with findings in GBM, by performing an immunohistochemical analysis in a cohort of 200 human TNBC samples, we found that the tumor cell expression of PDGFRβ marks a restricted subgroup of tumors (5.5% of total cases) with invasive and mesenchymal/stem-like phenotype [59]. Importantly, the use in both cellular and in vivo settings of the anti-PDGFRβ Gint4.T aptamer uncovered a still unappreciated role for PDGFRβ in driving ML TNBC cell invasiveness and metastases formation. Indeed, NIR-labeled Gint4.T, systemically intravenously administrated to mice bearing MDA-MB-231 xenografts, showed high tumor specificity, rapid tumor uptake (~2% injected dose at 15 min) and durable tumor retention (at least 24 h). Further, it revealed to be an efficacious probe to image, by 3D non-invasive fluorescence molecular tomography, the formation of lung metastases, obtained through the tail vein injection of MDA-MB-231 cells into nude mice. Notably, NIR-Gint4.T detected the metastatic suppression caused by the administration at a low therapeutic dose (eight treatments at ~0.74 mg aptamer/kg mouse body-weight) of the unlabeled aptamer [59], thus indicating Gint4.T as the first agent for molecular targeted noninvasive imaging and suppression of metastatic TNBC in the lungs.

### 5.2. Mucin 1

Mucin 1 (MUC1) is a highly glycosylated transmembrane protein expressed on the apical surface of most secretory epithelial cells where it provides a barrier between the cells and the environment. It has been found aberrantly glycosylated and overexpressed in a variety of epithelial cancers, including TNBC, with a clear role in enhancing invasiveness, metastasis, and resistance to reactive oxygen species [64]. Further, recent evidences suggest a role for MUC1 in metabolomic reprogramming of glutamine utilization in TNBC, which influences tumor growth [65]. Still, Burstein et al. reported MUC1 as predominantly expressed in the LAR subtype of TNBC [66].

To date, three different approaches targeting MUC1 (mAbs, vaccines and small peptide inhibitors of MUC1 cytoplasmic domain) have been developed and are in different phases of clinical trials for the treatment of human cancers, including breast cancers. Furthermore, a variety of anti-MUC1 mAbs have been successfully radiolabeled and used for tumor imaging [64]. As alternatives or complements to conventional antibodies, several anti-MUC1 aptamers have been developed. The DNA aptamers generated by Ferreira at al. [67] have been functionalized with commercially available chelators, radiolabeled with Technetium-99m (^99m^Tc) and widely employed for imaging modalities in a monomeric form or as multimeric conjugates [68,69,70]. More recently, these aptamers have been used to obtain early diagnosis of MUC1 overexpression in TNBC. Specifically, poly (lactic acid-*co*-l-acid) (PLGA) NPs, loaded with the anti-MUC1 aptamer and radiolabeled with ^99m^Tc, were administrated by retro-orbital injection to mice bearing subcutaneous MDA-MB-231 xenografts and animals were imaged by single photon emission computed tomography (SPECT). Even if the aptamer-based nanovectors were largely captured by the intestine, an uptake of 5% was observed in the tumor at 90 min after injection [71]. A great performance in terms of tumor accumulation (20% value at 90 min) was also obtained by using radiolabeled MUC1 aptamer-capped mesoporous silica NPs in the same animal model of TNBC [72]. Further, MUC1 aptamer was used as TNBC targeting agent in the construction of nanoprobes to be used for Raman imaging modalities. In this approach, surface-enhanced Raman spectroscopy (SERS) nanoprobes, made up of gold NPs labeled with infrared (IR) dyes (IR780 or IR792 perchlorate) as Raman reporter molecules, were coated with silica and functionalized with MUC1 aptamer. The nanoprobes, with or without aptamer functionalization, were intravenously injected into mice bearing tumors derived from two TNBC cell lines expressing MUC1 at a high and low extent, and the major tumor uptake was obtained by active targeting of MUC1 with a very low passive targeting [73]. Apart from the above aptamers, Ferreira et al. identified, with a second selection scheme, another DNA aptamer against MUC1, named 5TR1 [74], which was later modified with a GC loop at its 3′ end, for allowing conjugation to doxorubicin, and used for inhibiting the growth of MUC1-positive TNBC-derived tumors [75]. Further, 5TR1 aptamer was used as targeting agent for delivering bifunctional graphene oxide nanocomplexes, conjugated on their surface with either the anti-vimentin NAS-24 aptamer or a FAM-labeled anti-cytochrome c aptamer, to MUC1-positive MDA-MB-231 and MCF-7 cells. Interestingly, 5TR1-NAS-24-nano-complex induced apoptosis to target cells thanks to the pro-apoptotic function of the vimentin aptamer, and the second nanocomplex was able to image this event thanks to the binding of fluorescent anti-cytochrome c aptamer to its target in apoptotic cells [76].

### 5.3. Nucleolin

An attractive target for TNBC therapy is NCL, a nucleocytoplasmic protein involved in several biological processes, such as ribosomal assembly, rRNA processing, mRNA stability and miRNAs biogenesis, strongly associated with tumor development and aggressiveness, which is selectively expressed on the surface of cancer cells, but not on their normal counterparts [77]. Importantly, the first aptamer which entered phase I/II clinical trials (ClinicalTrials.gov identifier NCT00881244, NCT00740441) for cancer treatment is the 26-mer guanine-rich oligonucleotide named AS1411 [78]. After high affinity binding to NCL, AS1411 efficiently internalizes into cancer cells causing pleiotropic antiproliferative effects by mechanisms that are still under investigation. Several preclinical studies examined the roles of AS1411 binding to NCL in TNBC cell lines and animal models. Soundararajan et al. demonstrated that AS1411 competes with bcl-2 mRNA for binding to NCL, thus destabilizing bcl-2 mRNA in breast cancer cells, including MDA-MB-231 [79]. In a subsequent study, Pichiorri et al. showed that intraperitoneal injection of AS1411 into mice bearing orthotopic tumors derived from MDA-MB-231 cells, reduced lung metastases by affecting the NCL-dependent processing of a specific group of miRNAs involved in breast cancer aggressiveness [80]. Another study reported that AS1411-treatment of several cancer cell lines, including MDA-MB-231 and MDA-MB-468 TNBC cells, caused a type of non-apoptotic cell death characterized by hyperstimulation of macropinocytosis, and suggested a novel role for NCL in limiting the activation of Rac1, a driver of macropinocytosis [81].

To increase the efficacy of AS1411 in vitro [82] and in animal models of TNBC [83], two independent groups conjugated the aptamer onto gold nanostructures in order to achieve higher aptamer stability and local AS1411 concentration. In addition, to improve aptamer’s activity, nanoformulations of AS1411 have been explored for delivery of therapeutic agents specifically to NCL-positive TNBC cells. Indeed, AS1411-targeted polymeric NPs, loaded with the chemotherapeutic agent vinorelbine, enhanced cytotoxicity in MDA-MB-231 compared with non-targeted NPs [84]. Still, AS1411 was annealed, via a specific ‘sticky’ end, to one of the arms of a four-armed holliday junction DNA nanoconstruct carrying three siRNAs (Akt1, MDM2 and survivin) as therapeutic payload. The resulting multifunctional nanoconstruct allowed the simultaneous delivery of these siRNAs in MDA-MB-231 cells, causing a rapid induction of apoptosis [85]. In a different approach, Wang et al. used cell-derived extracellular vesicles (EVs), conjugated through cholesterol with AS1411, as a targeted delivery system for therapeutic let-7 miRNA or vascular endothelial growth factor siRNA, thus exploiting the advantages of natural carriers, such as low immunogenicity and long circulating times. The AS1411-EVs, loaded with let-7 miRNA and intravenously injected into mice bearing NCL-positive TNBC, inhibited tumor growth without nonspecific side effects or immune response [86].

## 6. Aptamers Targeting Surface Markers of Cancer stem Cells to Address TNBC Chemoresistance

Compared with other subtypes of breast cancer, TNBC has higher response rates to NAC, but paradoxically, after an initial response to treatment, patients commonly develop recurrent tumors, which are resistant to therapy and highly metastatic [1].

Recent evidence suggests that a small chemotherapy-resistant population of CSCs are the main cause of tumor relapse, thanks to their tumor-initiating as well as mesenchymal features [87]. CSCs, present at a significantly higher proportion in TNBC than other breast cancer subtypes (84.0% and 39.5% in TNBC and non-TNBC, respectively) [88], are intrinsically resistant to conventional chemotherapy. Indeed, they display properties closely related to drug resistance, including: (i) increased expression of drug efflux transporters; (ii) increased DNA repair response; (iii) activation of anti-apoptotic signaling pathways; (iv) overexpression of detoxifying enzymes [89,90]. Such features of CSCs allow them to survive treatment and spread to distant sites accounting for the aggressive phenotype of TNBC. Some evidences indicate that cytotoxic chemotherapy enhances tumor recurrence by induction in TNBC cells of pro-tumorigenic factors, including TGFβ, interleukin 6 (IL-6), IL-8, IL-1β, tumor necrosis factor-α and granulocyte-macrophage colony-stimulating factor (GM-CSF), which stimulate CSCs expansion [91,92]. It has been recently reported that paclitaxel enhances RNase activity of an endoplasmic reticulum stress sensor, IRE1, and this contributes to the secretion of pro-tumorigenic factors and ultimately to paclitaxel-mediated expansion of CSCs [92]. These findings suggest that the inclusion in TNBC therapeutic regimen of cytokines pathway and IRE1 RNase inhibitors can enhance the effectiveness of current chemotherapeutics. In addition, the hypoxia induced by chemotherapy provides another mechanism that contributes to the enrichment of the CSC population and, consequently, drug resistance and metastasis formation in TNBC [93]. Therefore, in light of the role of CSCs in clinical relapse and metastasis, it is imperative to develop new therapeutic strategies specifically targeting CSCs in order to achieve better treatment outcomes.

Currently, because CSCs carry surface markers such as CD44, aldehyde dehydrogenase 1, EpCAM, ATP-binding cassette sub-family G member 2 and CD133 [94], the use of mAbs that specifically recognize these biomarkers is the main approach to target these cells. Furthermore, several agents and strategies targeting CSC signaling pathways have been developed and entered in different phases of clinical trials. However, these CSCs-targeting molecules faced several obstacles such as poor tumor penetration, accumulation and high toxicity, which greatly hamper their clinical application [95]. Thus, aptamers represent valid alternative tools to current agents for overcoming resistance to treatments and tumor recurrence.

Noteworthy, cell-based selection of aptamers has a great potential to identify novel molecular targets on the surfaces of live CSCs [96,97]. In addition, several aptamers targeting specific CSC surface markers have been to date generated and used in preclinical studies as recently reviewed in [98]. Among these, some have been generated to potentially fulfill major obstacles in TNBC management (Figure 2).

The CD44 receptor has been identified as a marker of CSCs in many types of tumors, including TNBC, where its association with the Janus kinase 2 (JAK2)/signal transducer and activator of transcription 3 (STAT3) signaling pathway may account for aggressive behavior and resistance to therapy [55]. The 2′F-Py modified RNA aptamer, named Apt1, which was selected against the human recombinant full-length CD44 protein, was proved to bind with high affinity to CD44-positive MDA-MB-231, MCF-7 and T47D breast cancer cells [99] and, when conjugated on the outer shell of liposomes, it efficiently drove their specific uptake into TNBC cells [100]. Recently, in the perspective of set up a delivery system for siRNAs inducing silencing of disease genes in tumors, the same authors succeeded in entrapping siRNAs silencing the luciferase (luc2) reporter gene into non-cationic polyethylene glycol (PEG)ylated-liposomes, by using protamine to condense siRNAs. Resulting siRNA-loaded liposomes, decorated with Apt1, resulted able of efficient gene silencing both in vitro and in MDA-MB-231 orthotopic xenograft mouse model [101].

EpCAM is one of the cell surface markers used for the identification of CSCs from various epithelial cancers, including breast cancer [102]. Currently, several aptamer-based approaches to target CSCs have been developed by using EpCAM as CSC marker [97]. Much work has been performed by Shigdar and coworkers who developed and optimized 2′F-Py RNA aptamers against EpCAM [103,104] as alternative targeting options to the current anti-EpCAM antibody therapy [105]. Importantly, they proved superior performance of aptamers over antibodies in cancer theranostics due to their superior tumor penetration and target accessibility, thanks to the small size of the aptamers, and longer retention in tumor sites via conjugation to PEG, the most common chemical modification supported by aptamers for resisting renal clearance [106]. Because of their exquisite affinity and specificity, the anti-EpCAM aptamers discriminated breast cancer xenograft tissues, in tissue staining experiments with fluorescently labeled aptamers, depending on the expression level of EpCAM on cell surface [104]. Still, aptamer-siRNA chimeras, consisting of the anti-EpCAM aptamer as the targeting agent and polo-like kinase 1 siRNA as the therapeutic cargo, actively functioned on TNBCs accordingly to EpCAM expression in the tumors [107]. Indeed, accordingly to the high expression of EpCAM in TNBC cells belonging to BL TNBC subtype and poor expression in TNBC cells belonging to ML TNBC subtype, the RNA chimeras selectively inhibited colony and mammosphere formation (a CSC functional in vitro assay) of HCC1954, HCC1806, HCC1937 BL TNBC cells, even more efficiently than paclitaxel, but were inactive against MDA-MB-231 and BT-549 ML TNBC cells. The functional effect of the aptamer-siRNA conjugates versus epithelial and not mesenchymal TNBC was further confirmed by selective inhibition of tumor formation from chimera-treated BL TNBC cells in nude mice. Further, BL TNBC xenograft regression was observed upon subcutaneous injection of the chimeras. Taken together, these findings prove a powerful approach for treating epithelial TNBC subtype.

## 7. Aptamers Targeting the Tumor Microenvironment in TNBC

The role of TME in solid tumors as well as malignant hematological disease is well accepted. TNBC is considered to feature a unique microenvironment, distinct from that of other breast cancer subtypes, whose complexity has revealed additional biological barriers hindering efficacy of chemotherapy. Diverse components of the TNBC microenvironment, including fibroblasts, mesenchymal stem cells (MSCs), macrophages, adipocytes, altered ECM, synergistically promote tumor growth, metastasis and resistance to therapy [108]. Treatments targeting TME cellular components have advantages consisting in the inhibition of cells that are genetically stable unlike tumor cells. Indeed, the capability of cancer cells to develop and accumulate several mutations during disease progression as well as in response to therapy can cause drug resistance and failure of treatments. Therefore, researchers have recently attempted to explore the use of aptamer-based strategies for TME targeting (Figure 2). As discussed below, several proteins expressed by stromal cell types within the TNBC TME, as well as components of ECM, have been explored as targets for aptamers in therapeutic and imaging approaches.

### 7.1. Mesenchymal Stem Cells

Multipotent MSCs from bone-marrow (BM) and adipose tissue can be recruited to the TME in response to chemokines, cytokines and growth factors secreted by cancer cells. Once the MSCs come into contact with the tumor site they are “educated” to evolve and differentiate in tumor-associated MSCs (TA-MSCs) and/or cancer associated fibroblasts (CAFs) [109]. Both these cell types contribute to promote a pro-metastatic phenotype inducing cancer cell proliferation, migration and invasion as well as epithelial-mesenchymal transition (EMT) and immune evasion. Recently, our group showed, using non-invasive imaging, that the homing of near infrared (NIR)-labeled BM-derived MSCs to TME in an orthotopic TNBC mouse xenograft model caused an enhancement of lung metastases [110]. Consistent with the crucial role of PDGFRβ signaling in the recruitment of MSCs into TME [111], we proved that the inhibition of PDGFRβ expressed on the surface of BM-MSCs, by using a specific 2′F-Py-RNA aptamer [42,63], hampered the recruitment of MSCs towards TNBC cell lines, their trans-differentiation into CAF-like cells and, in vivo, their homing to TNBC xenografts and promotion of lung metastases [110]. These findings reveal aptamers as important tools to interrupt the bi-directional communications between cancer cells, MSCs and the inflammatory TME, which contribute to malignant tissue progression. In this context, aptamers were used as cell-surface sensors for probing in real-time the cellular niche environment and signaling. Specifically, fluorescent aptamers recognizing the PDGF were covalently attached onto the membrane of MSCs to detect PDGF secreted by neighboring MDA-MB-231 cells. Importantly, aptamer-MSCs retained their homing ability to BM and were monitored at the single-cell level by intravital microscopy 24 h post-transplantation into mice [112].

### 7.2. Vascular Endothelial Cells

Targeted delivery of therapeutics and imaging probes to the vasculature hold promise to improve management of many diseases, including cancer. The changes in the expression patterns of endothelial cell surface molecules in tumor-associated conditions, such as inflammation and neo-angiogenesis, renders these molecules suitable targets for selective delivery strategies. Aptamers are attractive compounds which can recognize the pathological vasculature. Within the selectin family, E-selectin represents a potential therapeutic target because it is highly expressed on endothelial cells of inflamed vessels but is absent in the normal vessels. Tanaka’s group reported the selection of a thiophosphate modified aptamer against E-selectin (ESTA) by using a SELEX approach in which, among the sequences selected against the human glycosylated recombinant E-selectin protein, the best ligands were screened by their ability to specifically bind to E-selectin-expressing human microvascular endothelial cells [113]. The ESTA aptamer recognized E-selectin with nanomolar affinity (Kd = 47 nM) on cultured endothelial cells and tumor-associated vasculature in human breast, ovarian, and skin carcinomas, and potently inhibited adhesion of a human promyelomonocytic cell line expressing sLex, a natural ligand for E-selectin, to E-selectin-expressing endothelial cells. A short while later, the same authors developed an ESTA-based approach to block E-selectin-supported hematogenous metastases of ER^−^/CD44^+^ breast cancer cells by preventing their adhesion to E-selectin-expressing premetastatic endothelial niche. They showed that a single intravenous injection of ESTA at a dose of 100 μg prevented the formation of lung metastasis of CD44-positive breast cancer cells intravenously injected in mice, both in a syngeneic (murine 4T1 cells) and xenogeneic (human MDA-MB-231 cells) model of breast cancer metastasis, without a relocation of metastases formation to a distinct site from lung upon aptamer treatment [114]. Further, the aptamer was truncated at its minimal variant necessary to obtain inhibition of the E-selectin/CD44 interaction, and the resulting short ESTA was conjugated with 10 kDa PEG for improving its pharmacokinetic parameters and anti-metastasis activity without causing tissue-damaging immune responses [115].

As a clear example of the functional versatility of aptamers, ESTA was evaluated not only as therapeutic agent but also as targeting ligand for drug delivery approaches. ESTA conjugation on the surface of rhodamine-tagged liposomes resulted in their accumulation at tumor vasculature at 5 h after intravenous injection into mice bearing MDA-MB-231 xenografts [116]. Still, as a proof-of-principle of a BM-targeted drug delivery strategy, ESTA was conjugated to biodegradable porous silicon microparticles (PSP) and showed to enhance the delivery of liposomes, incorporated into the porous structure of PSP, to the BM vasculature [117]. More recently, Mai et al. demonstrated the therapeutic efficacy of STAT3 siRNA, which was packaged into polymers loaded within the pores of PSP, on bone metastasis generated by intracardiac inoculation of MDA-MB-231 cells in mice [118].

### 7.3. Immune Cells

The heterogeneity of TNBC also extends to the tumor immune microenvironment, which displays substantial tumor-infiltrating lymphocytes (TILs), actively engaged in the process of ‘immunoediting’ [2]. The immune-checkpoint receptor PD-1 is expressed on TILs with the role of inhibiting effector T-cells, thus preventing autoimmunity and inflammatory response. Importantly, PD-L1, the PD-1 ligand, is highly expressed on the surface of TNBC cells and its interaction with PD-1 represents a common mechanism of tumor escape from immune destruction [4], thus providing the rationale for targeting the PD-1/PD-L1 axis in TNBC. Several clinical trials with immune checkpoint inhibitors, including mAbs targeting PD-1 (e.g., Nivolumab and pembrolizumab) and PDL-1 (e.g., atezolizumab, avelumab and durvalumab), are still at their beginning in TNBC. However, despite the promising results of their clinical efficacy as single agents as well as in the combination with various chemotherapeutics and radiation, challenges remain to improve rate and durability of responses, as well as overall survival [119]. Thus, an area of intense research is finding novel immunotherapy approaches in order to enlarge the repertoire of pharmacological strategies and overcome their limited efficacy.

Prodeus et al. developed a DNA aptamer, named MP7, able to bind at nanomolar affinity to the extracellular domain of murine PD-1 and suppress IL-2 secretion in primary T-cells [120]. The aptamer was conjugated at 5′ extremity with 40 kDa PEG to increase its in vivo half-life and successfully applied to suppress PD-1:PD-L1 interaction and growth of PD-L1-positive colon carcinoma cells in vivo, displaying a comparable efficacy to an antagonistic anti-PD-1 antibody. Furthermore, a DNA aptamer targeting human PD-L1 (aptPD-L1) was able to block the binding between human PD-1 and PD-L1 and helped T cell function restoration, modified TME and inhibited tumor growth in CT26 colorectal cancer and LL/2 lung cancer murine syngeneic tumor models [121]. Thus, even if not yet applied to TNBC, these studies suggest aptamers as a valid alternative to current antibody-based strategy in cancer immunotherapy.

More recently, Liu et al. [36] screened a DNA thioaptamer library in vivo by intravenous injection into mice bearing MDA-MB-231 breast cancer bone metastases. Following ten rounds of selection, a sequence (named T1) was identified able to accumulate in TME of breast tumors and bind with high affinity both polymorphonuclear myeloid-derived suppressor cells (PMN-MDSCs) and multiple breast cancer cells including TNBC cells. In an orthotopic breast cancer model the administration of liposomes, conjugated on the outer shell with T1 and loaded with doxorubicin, caused cancer cell apoptosis and a dramatic decrease of intratumoral population of MDSCs, thus increasing tumor infiltration of cytotoxic CD8^+^ T cells and, consequently, further cancer cell death.

### 7.4. Extracellular Matrix

Among the components of the ECM, osteopontin (OPN) is a phosphoprotein secreted by malignant cells in advanced metastatic cancer. It signals through αvβ3 integrin and CD44 adhesion molecules to increase invasion and metastases and to induce tumor-associated inflammatory cells and expression of angiogenic factors [122]. A nuclease-resistant 2′F-Py-RNA aptamer targeting OPN, termed OPN-R3, was generated that blocked the binding of OPN to CD44 and αvβ3 integrin receptors expressed on MDA-MB-231 cells, thus inhibiting OPN-dependent signal transduction pathways and decreasing expression levels of mediators of ECM degradation. This in turn resulted in inhibition of cell adhesion, migration and invasion in vitro [123]. In order to increase its biological half-life for in vivo applications, the aptamer was subject to 2′-*O*-methylation, 5′-cholesterol and 3′-inverted deoxythymidine modifications. When intravenously injected into mice bearing MDA-MB-231 orthotopic xenografts, the improved aptamer efficiently reduced local tumor growth and lung metastases formation [123]. Also, the modified aptamer showed adequate pharmacokinetic stability (7.8-h half-life in mouse serum) for its potential clinical application [124]. According to the inhibition of tumor growth and metastasis in the TNBC mouse model, a microarray analysis using RNA extracted from primary tumor from either untreated or aptamer-treated mice demonstrated that blocking OPN binding to cell surface receptors significantly altered the expression of genes critical for local tumor progression and metastases [125].

It has been reported that OPN produced by MDA-MB-231 cells causes a transformation of MSCs into CAFs through the MZF1 (myeloid zinc finger 1)-TGFβ1 pathway [126]. The inactivation of extracellular OPN by the OPN-R3 aptamer is able to inhibit TGFβ1-mediated MSC-to-CAF transformation and tumor growth and metastasis in a TNBC animal model [126]. More recently, the OPN aptamer was also proved efficacious to inhibit OPN-dependent migration of MSCs to the BM in orthotopic MDA-MB-231 and MCF-7 murine xenograft models, thus counteracting their action in enhancing the stemness phenotype of tumor cells in the BM premetastatic niche [127].

A capillary electrophoresis SELEX approach coupled to NGS (CE-NGS-SELEX) was developed in order to generate aptamers able to bind to ECM protein vitronectin (VN), used as target of the selection, and differentiate between VN and fibronectin (FN), a structurally related protein, used in the negative selection [128]. The VBA-01 aptamer coming out from the selection was successfully used for binding to human breast cancer tissues upon conjugation at its 3′ extremity to biotin and detection by using an anti-biotin linked horseradish peroxidase antibody. Further, when covalently linked to doxorubicin, the complex was proved efficacious to induce cytotoxicity to MDA-MB-231 cells at higher extent when they were cultured on VN-coated rather than on FN-coated plates, thus indicating the potential of the VBA-01 aptamer to improve drug delivery for breast cancer treatment.

Several studies have evidenced the role of the urokinase plasminogen activator (uPA) system, consisting of uPA, the uPA receptor (uPAR) and the plasminogen activator inhibitor (PAI-1), in regulating the cells-ECM interactions [129]. Both uPA and PAI-1 are markers of poor prognosis and metastases in primary breast tumors. The active form of the serine protease uPA converts plasminogen into plasmin, which in turn degrades the ECM directly or through the activation of pro-matrix metalloproteinases, thus promoting cancer cell metastasis and invasion. Moreover, uPAR binding to integrins, through the interaction with VN, regulates a variety of cellular processes, such as invasion, angiogenesis, and EMT. PAI-1 competes with integrins and uPAR on the surface of breast cancer cells for binding to VN causing detachment of cells from the ECM, which is critical to the metastatic process. With the intent to block the interaction between PAI-1 and VN, 2′F-Py RNA aptamers were isolated against PAI-1 that prevented the detachment of MDA-MB-231 cells from VN in the presence of PAI-1, resulting in an increase in cellular adhesion [130]. Later, the feasibility to express functional aptamers inside TNBC and endothelial cells by transfection of PAI-1 specific aptamers was demonstrated [131]. Anti-PAI-1 aptamer-expressing TNBC cells showed a decrease in cell migration and invasion thus indicating the involvement of intracellular PAI-1 in cancer progression.

## 8. Conclusions

In the era of precision medicine, currently cytotoxic chemotherapy represents the sole option to treat a TNBC. Aptamers represent a potential alternative option, but it is clear that their application as anticancer drugs are still limited, with only two aptamers, AS1411 (ClinicalTrials.gov identifier NCT00881244, NCT00740441) and NOX-A12 (ClinicalTrials.gov identifier NCT03168139), actually under clinical evaluation. Nevertheless, researchers worldwide are attempting to generate aptamers specifically targeting disease-related proteins and to develop them as targeted imaging agents, therapeutics and delivery agents for the management of a variety of human cancers, including TNBC. Several key articles have recently appeared, as discussed above, indicating that this is a rapidly evolving field. The exquisite targeting efficacy of aptamers, combined to their small size, ready chemical production, and a structure prone to manipulation for improving activity, overcoming nuclease susceptibility and resisting renal clearance, suggests that the practical usage of aptamers in cancer is not so far. In this respect, it is reasonable to predict that overcoming the major obstacles associated to TNBC management, such as the absence of specific biomarkers for a targeted diagnosis and therapy and their aggressive behavior may be achieved in a near future by the rational design of aptamer-based strategies.
